# Rapid inference of antibiotic resistance and susceptibility by genomic neighbour typing

**DOI:** 10.1038/s41564-019-0656-6

**Published:** 2020-02-10

**Authors:** Karel Břinda, Alanna Callendrello, Kevin C. Ma, Derek R. MacFadden, Themoula Charalampous, Robyn S. Lee, Lauren Cowley, Crista B. Wadsworth, Yonatan H. Grad, Gregory Kucherov, Justin O’Grady, Michael Baym, William P. Hanage

**Affiliations:** 1 Center for Communicable Disease Dynamics, Department of Epidemiology, Harvard T. H. Chan School of Public Health, Boston, MA USA; 2 Department of Biomedical Informatics and Laboratory of Systems Pharmacology, Harvard Medical School, Boston, MA USA; 3 Department of Immunology and Infectious Diseases, Harvard T. H. Chan School of Public Health, Boston, MA USA; 4 Division of Infectious Diseases, Department of Medicine, University of Toronto, Toronto, Ontario Canada; 5 Norwich Medical School, University of East Anglia, Norwich Research Park, Norwich, UK; 6 Epidemiology Division, Dalla Lana School of Public Health, University of Toronto, Toronto, Ontario Canada; 7 Department of Biology and Biochemistry, University of Bath, Bath, UK; 8 Thomas H. Gosnell School of Life Sciences, Rochester Institute of Technology, Rochester, NY USA; 9 CNRS/LIGM Université Paris-Est, Marne-la-Vallée, France; 10 Skolkovo Institute of Science and Technology, Moscow, Russia; 11 Quadram Institute Bioscience, Norwich Research Park, Norwich, UK

**Keywords:** Antimicrobial resistance, Pathogens, Sequencing, Medical research

## Abstract

Surveillance of drug-resistant bacteria is essential for healthcare providers to deliver effective empirical antibiotic therapy. However, traditional molecular epidemiology does not typically occur on a timescale that could affect patient treatment and outcomes. Here, we present a method called ‘genomic neighbour typing’ for inferring the phenotype of a bacterial sample by identifying its closest relatives in a database of genomes with metadata. We show that this technique can infer antibiotic susceptibility and resistance for both *Streptococcus pneumoniae* and *Neisseria gonorrhoeae*. We implemented this with rapid *k*-mer matching, which, when used on Oxford Nanopore MinION data, can run in real time. This resulted in the determination of resistance within 10 min (91% sensitivity and 100% specificity for *S. pneumoniae* and 81% sensitivity and 100% specificity for *N. gonorrhoeae* from isolates with a representative database) of starting sequencing, and within 4 h of sample collection (75% sensitivity and 100% specificity for *S. pneumoniae*) for clinical metagenomic sputum samples. This flexible approach has wide application for pathogen surveillance and may be used to greatly accelerate appropriate empirical antibiotic treatment.

## Abstract (web summary)

Genomic neighbour typing can be used to infer the antimicrobial susceptibility and resistance of a bacterial sample based on the genomes of closest relatives. Combined with MinION sequencing, it can rapidly determine microbial resistance for clinical samples within 4 h.

## Main

Infections pose multiple challenges to healthcare systems and contribute to higher mortality, morbidity and escalating cost. Clinicians must regularly make rapid decisions on empirical antibiotic treatment of infectious syndromes without knowing the causative pathogen (or pathogens) or whether they are drug-susceptible or drug-resistant. In some cases, this is directly linked to poor outcomes; in the case of septic shock, the risk of death increases by an estimated 10% with every 60 min of delay in initiating effective treatment^1^.

The molecular epidemiology of infectious disease allows us to identify high-risk pathogens and to determine their patterns of spread on the basis of their genetics or (increasingly) genomics. Conventionally, such studies, including outbreak investigations and characterization of previously untested resistant strains, have been conducted in retrospect, but this has been changing with the availability of increasingly inexpensive sequencing technologies^2,3^. The wealth of data generated by genomics are promising, but introduces a challenge because while many features of a sequence are correlated with the phenotype of interest, few are causative.

Prescription, however, has long been informed by correlative features when causative ones are difficult to measure; for example, whether the same syndrome or pathogen occurring in other patients from the same clinical environment have responded to a particular antibiotic. This has also been observed at the genetic level as a result of genetic linkage between resistance elements and the rest of the genome. An example is given by the pneumococcus *S*. *pneumoniae*. The Centers for Disease Control and Prevention (CDC) has rated the threat level of drug-resistant pneumococcus as “serious”^4^. While resistance arises in pneumococci through a variety of mechanisms, approximately 90% of the variance in the minimal inhibitory concentration (MIC) for antibiotics of different classes can be explained by the loci determining the strain type^5^, even though none of these loci themselves causes resistance. Thus, in the overwhelming majority of cases, resistance and susceptibility can be inferred from coarse strain typing based on the population structure. This population structure could be leveraged to offer an alternative approach to detecting resistance whereby rather than detecting high-risk genes, we identify high-risk strains. While many approaches have been developed to identify whether a pathogen carries mutations or genes known to confer resistance^6–21^ (see ref. ^22^ for a comprehensive review), this is not equivalent to the clinical question of whether the pathogen is susceptible.

We present a method called genomic neighbour typing that can bring molecular epidemiology closer to the bedside and provide information relevant to treatment at a much earlier stage. Our method takes sequences generated from a sample in real time and matches them to a database of genomes to identify the closest relatives. Because closely related isolates usually have similar properties, this yields an informed heuristic regarding the phenotype of the pathogen. We demonstrate this by identifying drug-resistant and drug-susceptible clones for both *S*. *pneumoniae* (the pneumococcus) and *N*. *gonorrhoeae* (the gonococcus) within minutes after the start of sequencing using Oxford Nanopore Technology (ONT). The method has many potential applications depending on the specific pathogen and the quality of the databases available for matching, which we discuss together with its limitations.

## Results

### Resistance is associated with clones in *S. pneumoniae* and *N. gonorrhoeae*

To quantify the association of clones with antibiotic resistance of the pathogens *S. pneumoniae* and *N. gonorrhoeae*, we constructed optimal predictors of resistance from bacterial lineages and measured the associated area under the receiver operating characteristic curve (AUC) (Supplementary Fig. 1). First, we applied the method to 616 pneumococcal genomes from a carriage study of children in Massachusetts, USA^23,24^. Second, we used 1,102 clinical gonococcal isolates collected from 2000 to 2013 by the CDC’s Gonococcal Isolate Surveillance Project^25^. In both cases, the datasets comprised draft genome assemblies from Illumina HiSeq reads, resistance data and lineages inferred from sequence clusters computed using Bayesian analysis of population structure^26^. Lineages of *S. pneumoniae* were predictive for benzylpenicillin, ceftriaxone, trimethoprim–sulfamethoxazole, erythromycin and tetracycline resistance, with AUC values ranging from 0.90 to 0.97 (Supplementary Fig. 2), which is consistent with previous works^5^. For *N. gonorrhoeae*, ciprofloxacin, ceftriaxone and cefixime attained comparably large AUC values (from 0.93 to 0.98), whereas azithromycin demonstrated a lower association (AUC value of 0.80) (Supplementary Fig. 3), which is as previously observed^25^.

### Rapid identification of nearest known relatives from sequencing reads

Based on the observed associations, we developed an approach that we term genomic neighbour typing to predict the phenotype from sequencing data. Genomic neighbour typing is a two-step algorithm that first compares a provided sample to a database of reference genomes with a known phylogeny and phenotype, and then predicts the probable phenotype of the sample based on the best hits (nearest neighbours) and their matching quality. We apply this here to the detection of drug resistance.

**Fig. 1:**
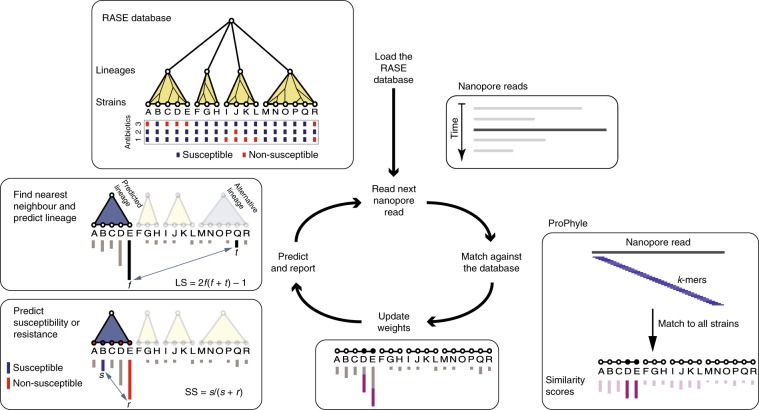
Overview of the RASE approach. In the first loading step, the precomputed RASE database is loaded into memory. As reads are generated, they are matched against the database using ProPhyle to calculate similarity to individual strains. The weights for the most similar strains (D and E in the figure) are increased proportionately to the number of matching *k*-mers. Finally, resistance is predicted from the obtained weights and from the resistance profiles of the database strains in the following manner. First, the best lineage is identified as the lineage of the best match (having the highest weight, E in the figure) and its score is calculated (lineage score (LS)). Second, for every antibiotic, a score quantifying the chance of susceptibility (susceptibility score (SS)) is calculated based on the most similar susceptible and resistant strains inside the identified lineage (B and E in the figure, respectively). The susceptibility or resistance to each of the antibiotics is predicted from their susceptibility scores by a comparison with a threshold (0.5 in the default setting) and reported together with the lineage, the best matching strain and the known properties of that strain (for example, the original antibiograms and the MLST-identified sequence type or serotype).

To implement genomic neighbour typing, we developed software called resistance-associated sequence elements (RASE) (Fig. 1). RASE takes a stream of nanopore reads and compares their *k*-mer content to references using a modified version of ProPhyle^27,28^, which is a metagenomic classifier that implements a fast and memory-efficient exact coloured de Bruijn graph data structure^29^ using a Burrows–Wheeler transform (BWT) index^30^ (Methods). Using ProPhyle RASE identifies which references are the most similar to the read and increases their similarity weights (this approach was inspired by but differs from other similar approaches such as Kraken^31^ and Kallisto^32^). These weights are cumulative scores that capture sample-to-reference similarity; they are set to zero at the beginning and are increased on the fly as sequencing proceeds according to the ‘information content’ of each read (Methods). Generally speaking, longer reads, such as those covering multiple accessory genes, tend to be specific and have high scores, whereas short reads or reads from the core genome are found in many lineages, tend to be nonspecific and have low scores. Weights serve as a proxy to the inverted genetic distance between the sample and the references.

Resistance or susceptibility is predicted in two steps based on the computed weights, the population structure and the reference phenotypes. First, RASE identifies the lineage of the best-matching reference genome and estimates the confidence of lineage assignment by comparing the two best-matching lineages to compute a ‘lineage score’ (Methods). Subsequently, RASE identifies the best match within that lineage and predicts resistance from the nearest resistant and susceptible neighbours. A comparison of their weights provides a ‘susceptibility score’, which quantifies the risk of resistance (Methods). When the weights are too similar, the confidence of the call is considered low; this happens when resistant and susceptible strains are insufficiently genetically distinct, which is often the case for resistance that has recently emerged in evolutionary history (Methods). The ability to pinpoint the closest relatives in the database offers further resolution, even in the case where the resistance phenotype varies within a lineage.

**Fig. 2:**
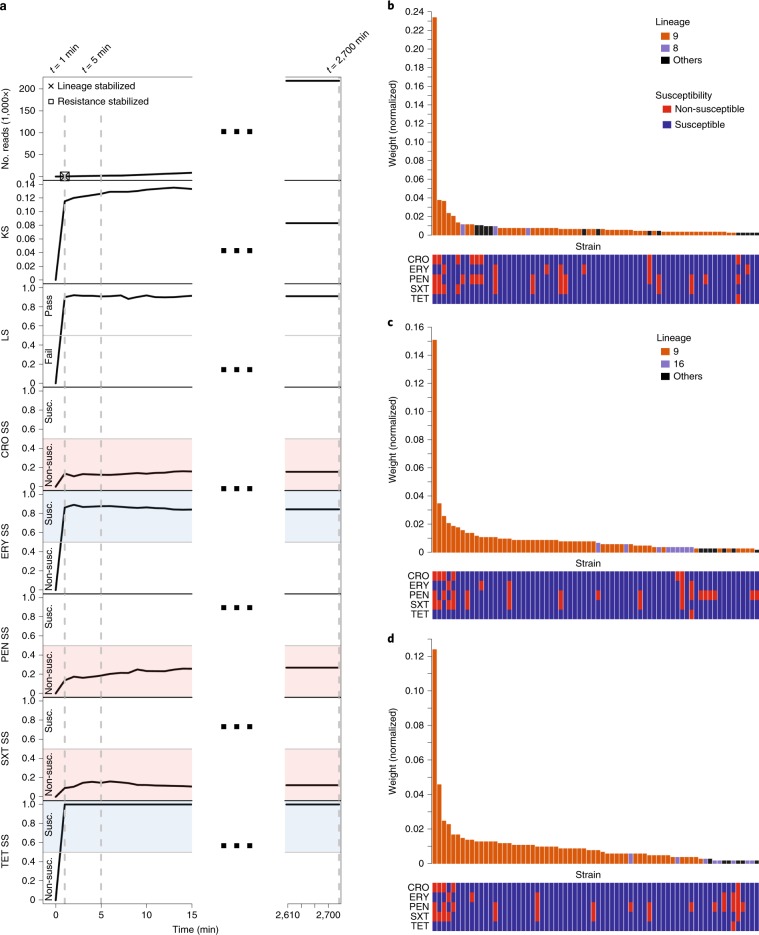
RASE obtains stable predictions of antibiotic resistance or susceptibility and lineage within minutes for an isolate of a pneumococcal 23F clone (SP06). **a**, The number of reads, LS, KS and SS for individual antibiotics as a function of time from the start of sequencing. In the top left plot, the times of stabilization are shown for the predicted lineage and susceptibility or resistance to all antibiotics (ceftriaxone (CRO), erythromycin (ERY), benzylpenicillin (PEN), trimethoprim–sulfamethoxazole (SXT) and tetracycline (TET)). Blue and red colours correspond to susceptibility (Susc.) and non-susceptibility (Non-susc.) calls, respectively. The dashed lines mark selected time points (1 min, 5 min and the end of sequencing (2,700 min)). **b**–**d**, Similarity rank plots for selected time points: 1 min (**b**), 5 min (**c**) and the end of sequencing (2,700 min; **d**). The bars correspond to the 70 best-matching strains in the database and display the normalized weights, which serve as a proxy to the inverted genetic distance. They are arranged by rank and coloured according to the presence in the predicted, alternative or another lineage. The panels underneath each chart display the resistance profiles of the strains.

Results of RASE are reported in real time as the best match in the database, together with susceptibility scores to the antibiotics being tested and a proportion of matching *k*-mers for quality control. As the run progresses, the scores fluctuate and eventually stabilize (an example is shown in Fig. 2).

### RASE databases for hundreds of *S. pneumoniae* and *N. gonorrhoeae* strains

**Extended Data Fig. 1:**
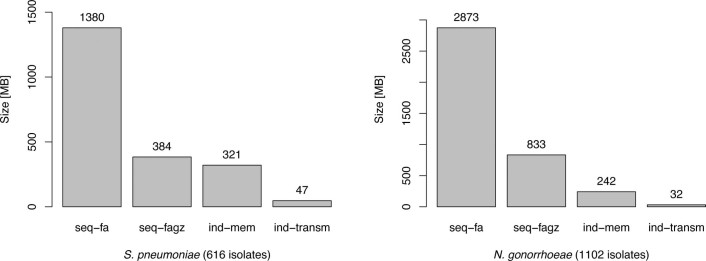
Size and memory footprint of the *S. pneumoniae* and *N. gonorrhoeae* RASE databases. The graph compares the size of the ProPhyle RASE index to the size of the original sequences: original draft assemblies (seq−fa), original draft assemblies compressed using gzip (seq−fagz), memory footprint of ProPhyle with the RASE index (ind−mem), and size of the ProPhyle RASE index compressed for transmission (ind−transm).

We constructed RASE databases for *S. pneumoniae* and *N. gonorrhoeae* from the same data as described above (Methods). We assigned each pneumococcal and gonococcal strains to antibiotic-specific resistance categories using the European Committee on Antimicrobial Susceptibility Testing (EUCAST) breakpoints^33^ and the CDC Gonococcal Isolate Surveillance Project (GISP) breakpoints^34^, respectively (Methods). Where MIC data were unavailable or insufficiently specific, we estimated the probable resistance phenotype using ancestral state reconstruction (Methods; Supplementary Note 1; Extended Data Figs. 3 and 4). To verify the results, we tested eight pneumococcal isolates for which resistance phenotypes were not originally available (Methods), and the measured MICs by microdilution matched the expected phenotypes (shown in bold in Table 1). We constructed the ProPhyle *k*-mer indexes using a *k*-mer length that was optimized to minimize prediction delays (*k* = 18; Methods). The obtained pneumococcal and gonococcal RASE databases occupy 321 MB and 242 MB of RAM (×4.3 and ×12, respectively, compression rate) and can be further compressed for transmission to 47 MB and 32 MB (×29 and ×90, respectively, compression rate), respectively (Extended Data Fig. 1). This would allow RASE to be used on portable devices and its databases to be easily transmitted to the point of care over links with a limited bandwidth.

**Table 1:** Predicted phenotypes of *S. pneumoniae* for database isolates (a), non-database isolates (b) and metagenomes (c)

(a) Database isolates																
Sample	Lineage confidently detected	Matched *k*-mers (%)	Serotype		Antibiogram CRO		Antibiogram ERY		Antibiogram PEN		Antibiogram SXT		Antibiogram TET		MLST match	CC match
			Actual	Best match	Actual	Best match	Actual	Best match	Actual	Best match	Actual	Best match	Actual	Best match		
SP01	Yes	16	11D	11D^a^	S	S^a^	S	S^a^	S	S^a^	S	S^a^	**S**^**(1)**^	**S**^**(1)**a^	Yes^a^	Yes^a^
SP02	Yes	9.6	19A	19A^a^	R	R^a^	R	R^a^	R	R^a^	R	R^a^	**R**^**(2)**^	**R**^**(2)**a^	Yes^a^	Yes^a^

(b) Non-database isolates																
Sample	Lineage confidently detected	Matched *k*-mers (%)	Serotype		Antibiogram CRO		Antibiogram ERY		Antibiogram PEN		Antibiogram SXT		Antibiogram TET		MLST match	CC match
			Actual	Best match	Actual	Best match	Actual	Best match	Actual	Best match	Actual	Best match	Actual	Best match		
SP03	Yes	3.1	23F	23F^a^	R	R^a^	R	**S**^**(3)**b^	R	R^a^	R	R^a^	S	S^a^	OoD^c^	Yes^a^
SP04	Yes	12	19A	19A^a^	R	R^a^	R	R^a^	R	R^a^	R	R^a^	R	**R**^**(4)**a^	OoD^c^	Yes^a^
SP05	No	1.8	19F	19F^a^	R	R^a^	R	R^a,d^	R	R^a^	R	R^a^^,d^	R	R^a,d^	OoD^c^	Yes^a^
SP06	Yes	8.3	23F	23F^a^	R	R^a^	R	**S**^**(3)**b^	R	R^a^	R	R^a^	S	S^a^	OoD^c^	Yes^a^

(c) Metagenomes																
Sample	Lineage confidently detected	SP (%)	Matched *k*-mers (%)		Antibiogram ERY				Antibiogram PEN				Antibiogram TET			
					Actual		Best match		Actual		Best match		Actual		Best match	
SP07	No	2.3	0.2		NA		S^c^		S		S^a^		R		**S**^**(5)**b^	
SP08	No	2.5	0.9		S		S^a^		S		S^a,d^		S		**S**^**(6)**a^	
SP09	No	4.0	1.2		NA		S^c^		S		S^a^		S		**S**^**(7)**a^	
SP10	Yes	21	5.2		R		R^a^		R		R^a^		R		**R**^**(8)**a^	
SP11	Yes	70	14		R		R^a^		R		R^a^		R		**R**^**(8)**a^	
SP12	Yes	86	17		S		S^a^		S		S^a^		R		**S**^**(5)**b^	

The table displays actual and predicted resistance phenotypes (where ‘S’ represents susceptible and ‘R’ represents non-susceptible) for individual experiments and information on the match of the predicted MLST-identified sequence type and the clonal complex (CC). Resistance categories in bold were inferred using ancestral reconstruction and were confirmed using phenotypic testing (see Methods and Supplementary Table 2). Metagenomic samples were sorted by the estimated fraction of *S. pneumoniae* reads (SP).

^a^Correct prediction.

^b^Incorrect prediction.

^c^Cannot be evaluated.

^d^Low confidence call. OoD, out of database; ^(*n*)^, identity of a retested sample; NA, not available. For definitions of antibiotics see Fig. 2.

### RASE identifies strains in the database within minutes

**Extended Data Fig. 2:**
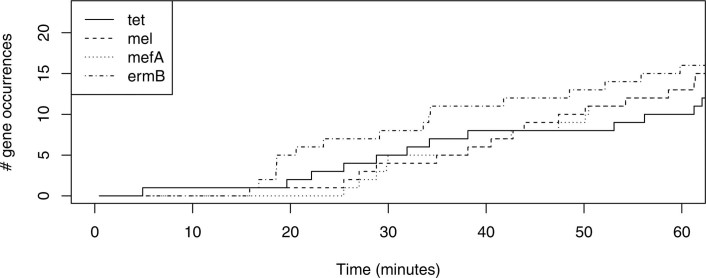
Timeline of resistance genes. Number of occurrences of individual resistance genes in reads of SP02, as a function of time for the first hour of nanopore sequencing.

We first examined two pneumococcal isolates that were used to build the RASE database (100% sensitivity and 100% specificity, *n* = 10; Table 1a) to test whether RASE can function in ideal circumstances. In the case of a fully susceptible isolate (SP01), the correct lineage and sequenced strain were identified within 1 min and 7 min, respectively. A multidrug-resistant isolate (SP02) was predicted even faster, with both lineage and the sequenced strain correctly detected and stabilized within 1 min. To compare our method to gene-based approaches for detecting resistance^22^, we evaluated how long it took for resistance genes to be sequenced on the device. We observed that at least 25 min would be needed for single copies to be detected (Supplementary Note 2; Extended Data Fig. 2).

We then performed a similar evaluation with five gonococcal isolates from the database (57% sensitivity and 100% specificity, *n* = 20; Table 2a); however, here, we selected more complicated cases. First, we tested a susceptible isolate (GC01), for which RASE identified the correct strain and antibiogram within 3 min of sequencing. We then sequenced an isolate (GC02) with an uncommon mechanism of cephalosporin resistance that has recently emerged^35^. Under such circumstances, the resistant strain and its susceptible neighbours tend to be genetically very similar, which could confound our analysis. However, RASE was still able to identify the correct resistance phenotypes in 9 min, with the delay due to the difficulty in distinguishing between the close relatives, which was reflected by a susceptibility score in the low-confidence range (Methods). This was repeated in further experiments with the same isolate (GC03), which consistently reported low confidence in the resistance phenotype (Methods). This feature of our approach intends to alert the operators to indicate that further testing is necessary. In this experiment, RASE also resolved sample mislabelling (Supplementary Note 3). For a multidrug-resistant isolate (GC04), RASE predictions stabilized within 2 min, but incorrectly predicted susceptibility to ceftriaxone. A subsequent analysis revealed that the ceftriaxone MIC of the sample was equal to the CDC GISP breakpoint (0.125 μg ml^–1^), whereas the best match in the database had an MIC of 0.062 μg ml^–1^, within a single doubling dilution. We further found that RASE performed well even with extremely poor data and low-quality reads (for example, GC05; Supplementary Note 4). We also evaluated how genomic neighbour typing would perform if RASE used Kraken^31^ instead of ProPhyle^27,28^, and results are presented in Supplementary Note 5.

**Table 2:** Predicted phenotypes of *N. gonorrhoeae* for database isolates (a) and non-database isolates (b)

(a) Database isolates																					
Sample	Lineage confidently detected		Matched *k*-mers (%)		Antibiogram AZM				Antibiogram CFM				Antibiogram CIP				Antibiogram CRO				MLST match
					Actual		Best match		Actual	Best match			Actual	Best match			Actual	Best match			
GC01	Yes		27		S		S^a^		S	S^a^			S	S^a^			S	S^a^			Yes^a^
GC02	Yes		27		S		S^a^		R	R^a,c^			S	S^a^			R	R^a,c^			Yes^a^
GC03	Yes		33		S		S^a^		R	S^b,c^			S	S^a^			R	S^b,c^			Yes^a^
GC04	Yes		21		S		S^a^		R	R^a^			R	R^a^			R	S^b^			Yes^a^
GC05	Yes		7		R		R^a^		S	S^a^			S	S^a^			S	S^a^			Yes^a^

(b) Non-database isolates																					
Sample		Lineage confidently detected		Matched *k*-mers (%)		Antibiogram AZM					Antibiogram CFM				Antibiogram CIP				Antibiogram CRO		
						Actual		Best match			Actual	Best match			Actual	Best match			Actual	Best match	
GC06		Yes		19		S		S^a^			R	R^a^			R	R^a^			S	S^a^	
GC07		No		20		S		S^a^			S	S^a^			R	R^a^			S	S^a^	
GC08		No		19		S		S^a^			S	S^a^			R	R^a^			S	S^a^	
GC09		No		18		S		S^a^			S	S^a^			S	S^a^			S	S^a^	
GC10		No		20		S		S^a^			S	S^a^			R	R^a^			S	S^a^	
GC11		No		20		S		S^a^			S	S^a^			R	R^a^			S	S^a^	
GC12		No		20		S		S^a^			S	S^a^			R	R^a^			S	S^a^	
GC13		Yes		20		S		S^a^			S	S^a^			R	R^a^			S	S^a^	
GC14		Yes		19		S		S^a^			S	S^a^			R	R^a^			S	S^a^	
GC15		Yes		19		R		S^b,c^			S	S^a^			S	S^a^			S	S^a^	
GC16		No		18		S		S^a^			S	S^a,c^			R	R^a^			S	S^a,c^	
GC17		No		19		S		S^a^			S	S^a,c^			R	R^a^			S	S^a,c^	
GC18		No		20		S		S^a^			S	S^a^			R	R^a^			S	S^a^	
GC19		Yes		18		S		S^a^			S	S^a^			R	R^a^			S	S^a^	

The table displays actual and predicted resistance phenotypes (S and R) for individual experiments and information on the match of the predicted MLST-identified sequence type.

^a^Correct prediction.

^b^Incorrect prediction.

^c^Low-confidence call. AZM, azithromycin; CFM, cefixime; CIP, ciprofloxacin.

### RASE identifies the closest relative of previously untested isolates

We next examined four pneumococcal isolates (89% sensitivity and 100% specificity, *n* = 20; Table 1b) for which the serotype and limited antibiogram and lineage data were known. We compared the following three characteristics of the sample to assess our performance: the serotype, the sequence type as determined by multilocus sequence typing (MLST) and the antibiograms (benzylpenicillin, ceftriaxone, trimethoprim–sulfamethoxazole, erythromycin and tetracycline resistance according to the EUCAST breakpoints^33^).

In all cases, the closest relative was identified within 5 min, even if the correct MLST sequence type was absent from the RASE database (an example is shown in Fig. 2). The two samples from the 23F clone (SP03 and SP06) were correctly called as being closely related to the Tennessee 23F-4 clone identified by the Pneumococcal Molecular Epidemiology Network, a clone strongly associated with macrolide resistance^36^. Consistent with this, the two samples were indeed resistant to erythromycin. However, the Tennessee 23F-4 clone was absent from the Massachusetts sample, with the best match being a comparatively distantly related strain that was resistant to penicillin, but susceptible to erythromycin. This illustrates the importance of a relevant database.

We evaluated RASE with 14 clinical gonococcal isolates from the RaDAR-Go project^37^ (Switzerland, 2015–2016) (93% sensitivity and 100% specificity, *n* = 56; Table 2b). These isolates were previously sequenced using nanopore and have full antibiograms available^38^. The 55 out of 56 correct calls indicate the strength of the genomic neighbour typing in a clinical setting. The only incorrect call (susceptibility to azithromycin for GC15) was marked as being low confidence on the basis of a poor susceptibility score. It should be noted that the ranges for what is considered low confidence could vary among settings and pathogens, but can be empirically determined and modified by users. In this case, our results suggest that informative results can be obtained even when using a database from one region (the United States) to predict phenotypes in another region (Europe). However, this may not be the case for all pathogens.

### Inference is still informative but lower quality on highly divergent lineages

As noted above, an important precondition of genomic neighbour typing is a comprehensive and relevant reference database. To evaluate the performance of RASE in a setting with an incomplete database, we used the gonococcal WHO (World Health Organization) 2016 reference strain collection^39^. This includes a global collection of 14 diverse isolates from Europe, Asia, North America and Australia, collected over two decades and exhibiting phenotypes ranging from pan-susceptibility to multidrug resistance, and as such, the GISP database is expected to be non-representative in this study. The WHO strains are available from the National Collection of Type Cultures, and were previously sequenced using nanopore^38^ and genetically and phenotypically characterized^39^. Surprisingly, RASE correctly identified all MLST-identified sequence types represented in the database, and in seven cases it provided fully correct resistance phenotypes (67% sensitivity and 91% specificity, *n* = 56; Supplementary Table 1). In six out of seven cases where the complete resistance profile was not recovered, the closest relatives were correctly identified, but were genetically divergent from the query isolates (Supplementary Note 6). In one case, the errors were due to a misidentification of the closest relatives by ProPhyle. Therefore, most prediction errors could be addressed with a more comprehensive database.

### RASE can identify resistance in pneumococcus from sputum metagenomic samples

Because bacterial culture and phenotyping via agar-dilution, Etest or disk diffusion introduces significant delays in resistance profiling, direct metagenomic sequencing of clinical samples would be preferable for point-of-care use. We therefore analysed metagenomic nanopore data from sputum samples obtained from patients suffering from lower respiratory tract infections^40^ (UK, 2017), selecting six samples from the study that were already known to contain *S. pneumoniae* (75% sensitivity and 100% specificity, *n* = 16; Table 1c).

One sample (SP10) contained DNA from multiple bacterial species. However, within 5 min, the sequence was identified to belong to the Swedish 15A-25 clone (ST63), which is also known to be associated with resistance phenotypes including macrolides and tetracyclines^41^. This sample was confirmed to be resistant to erythromycin and to clindamycin, tetracycline and oxacillin according to the EUCAST breakpoints^33^. The original report of the Swedish 15A-25 clone did not report resistance to penicillin antibiotics^41^, which has subsequently emerged in this lineage. However, our database correctly identified the risk of penicillin resistance in this sample. The metagenomes SP11 and SP12 contained an estimated >20% reads that matched *S. pneumoniae*, and their serotypes were identified to be 15A and 153, respectively. The susceptibility scores of the best matches were fully consistent with the resistance profiles found in the samples, with the exception of tetracycline resistance in SP12 due to an incomplete database (Supplementary Note 7). The last remaining samples, SP07–SP09, contained <5% unambiguously pneumococcal reads. Despite the low proportions, all predicted phenotypes were concordant with phenotypic tests, with the exception of SP07, which matched the same strain as SP12 (discussed above).

## Discussion

This paper presents a method, which we termed genomic neighbour typing, to pinpoint the closest relatives of a query genome within a suitable database and then to infer the phenotypic properties of the query strain on the basis of the reported properties of its relatives. At present, the precise lineage of a bacterial pathogen is often determined after most important clinical decisions have been made. However, incorporating genomic neighbour typing at an earlier stage offers a way of leveraging bacterial population structure to gain information on resistance and susceptibility, and to guide antimicrobial therapy. The results from the metagenomic samples suggest that it is possible to apply this approach directly to clinical samples, and the success with both *S. pneumoniae* and *N. gonorrhoeae* indicates that it may have wide application.

The two pathogens studied here present contrasting features; the gonococcus is Gram-negative, harbours plasmids and has a strikingly uniform core genome, while the pneumococcus is Gram-positive, does not contain plasmids and is diverse in both its core and accessory genome. Both exhibit high rates of homologous recombination, which is expected to both spread chromosomally encoded resistance elements and to scramble the phylogenetic signal that we use to identify the lineages. Despite these differences and the large degree of recombination, our approach performs well with both pathogens, with some differences that indicate opportunities and limitations of the application.

The initial identification of the closest relative was consistently more robust in the pneumococcus than the gonococcus, which is a result of the former having more *k*-mers that are specific to an individual lineage, thus reflecting greater sequence diversity. As a consequence of the much lower diversity in gonococcus, when multiple closely related genomes are present in the database, RASE fluctuated between them, even though it correctly identified the region of the phylogeny. If these genomes vary in their resistance profile, this is properly reflected in an uncertain susceptibility score that indicates caution and further investigation are merited (for example, GC03).

As in all inference, the principle limitation of genomic neighbour typing is the representativeness of the database. While we have made use of relatively small samples from limited geographical areas to demonstrate proof of principle, in practice, there are multiple examples of large genomic databases generated by public health agencies, which could be combined with metadata on resistance for genomic neighbour typing. Such databases could, if necessary, be supplemented with local sampling. The relevant question for our approach therefore becomes whether the database contains a sufficiently high proportion of strains that will be encountered in the clinic and whether the resistance data are correct. Further work is required to determine the optimal structure and contents of databases for each application, but we emphasize the range of pathogens that appear to show promise for this approach. These include *Escherichia coli*, in which data on MLST-identified sequence types supplemented with epidemiological information consistently produced AUC values in excess of 0.90 for multiple antibiotics^42^, which suggests that there is great potential for neighbour typing to offer excellent resolution that is superior to MLST. However, we anticipate that genomic neighbour typing may be less suitable in cases when there is little within-species genomic variation (meaning it is hard to identify the nearest neighbour) or when resistance rapidly emerges on independent and diverse genomic backgrounds (meaning resistance is poorly correlated with those backgrounds) (Supplementary Note 8).

In cases where the infectious agent is unknown, this problem is significantly more challenging. *k*-mers from one pathogen can match others and produce false predictions, and so the choice of the correct database for prediction is key. Doing this will probably require a two-step solution in which the reads are first passed through a metagenomic classifier such as Centrifuge^43^ or MetaMaps^44^, which would be used to select the correct RASE database on which to make a resistance call.

Another limitation is the time required for sample preparation, which currently includes human DNA depletion, DNA isolation and library preparation, thereby taking a total of 4 h. However, this is a rapidly evolving area of technology and automated rapid library preparation kits are already in development^45^. Further advances in this space, in particular for the preparation of metagenomic samples, will be required to bring the method closer to the bedside.

We have demonstrated that effectively predicting resistance and susceptibility from sequencing data does not require knowledge of causal resistance determinants. In fact, neighbour typing only requires that the phenotype be sufficiently strongly associated with the population structure to make reliable predictions.

A key advantage of this approach is that it requires very little genomic data, thus it is not limited by high error rates or low coverage. In particular, it is not attempting to define the exact genome sequence of the sample being tested, but merely which lineage it comes from. As a result, even when a small fraction of *k*-mers in the read are informative in matching to the RASE database, this is sufficient to call the lineage. This has the benefit of being faster than gene detection by virtue of the informative *k*-mers being distributed throughout the genome, and so more likely to appear in the first few reads sequenced by the nanopore. Therefore, the approach we present here can be seen as an application of compressed sensing; that is, by measuring a sparse signal distributed broadly across our data, we can identify it with comparatively few error-tolerant measurements.

Genomic neighbour typing can also be used to detect other phenotypes that are sufficiently tightly linked to a phylogeny, such as virulence. Further applications may include rapid outbreak investigations, as the closely related isolates involved in the outbreak would all be predicted to match to the same strain in the RASE database. The approach also lends itself to enhanced surveillance, including in the field; the 2014–2016 Ebola outbreak in West Africa, for example, saw MinION devices used in remote locations without advanced healthcare facilities^2^. Finally, at present, empirical treatment decisions are made within successive ‘windows’^46^, in which increasing information becomes available, from initial Gram stain to full phenotypic characterization. The information from genomic neighbour typing is a natural complement to this process and has the potential to improve therapy long before it would become clinically apparent that the patient is not responding or before phenotypic susceptibility data were available. The combination of high-quality RASE databases with genomic neighbour typing offers an alternative forward-looking model for diagnostics and surveillance, with wide applications for the improved clinical management of infectious disease.

## Methods

### Overview

RASE uses rapid approximate *k*-mer-based matching of long sequencing reads against a database of strains to predict resistance via neighbour typing. The database contains a highly compressed exact *k*-mer index, a representation of the tree population structure and metadata such as lineage, resistance profiles, MLST-based sequence type and serotype. The RASE prediction pipeline iterates over reads from the nanopore sequencer and provides real-time predictions of lineage and resistance or susceptibility (Fig. 1).

### Resistance profiles

**Extended Data Fig. 3:**
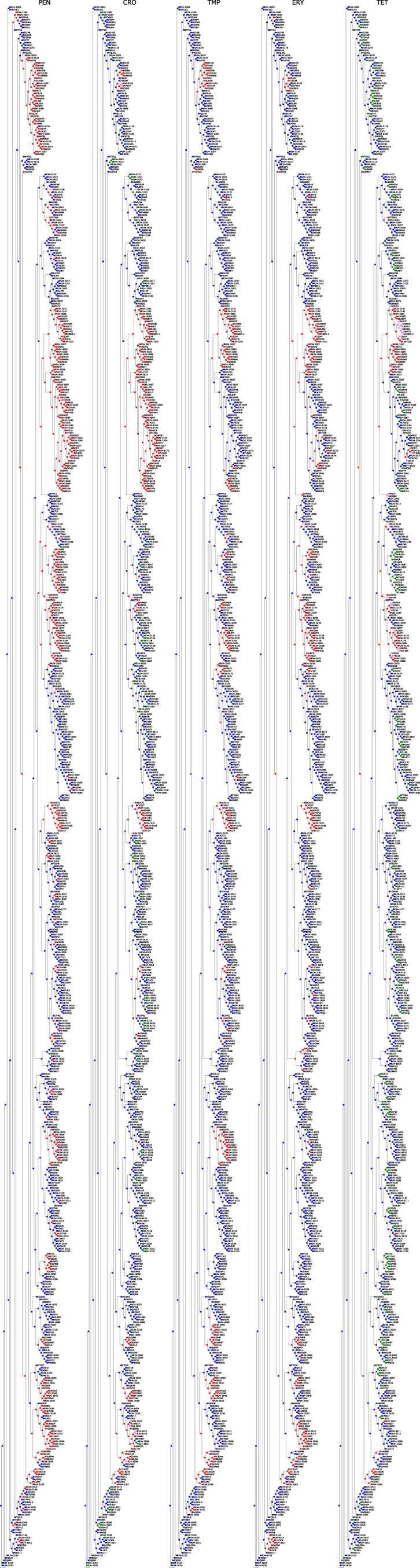
Ancestral state reconstruction of resistance categories in the *S. pneumoniae* RASE database. Each panel corresponds to a single antibiotic and displays the database phylogenetic tree, colored according to the reconstructed resistance categories for the antibiotic (blue, green, red, violet correspond to ‘susceptible’, ‘unknown – inferred susceptible’, ‘non-susceptible’, ‘unknown – inferred non-susceptible’, respectively).

**Extended Data Fig. 4:**
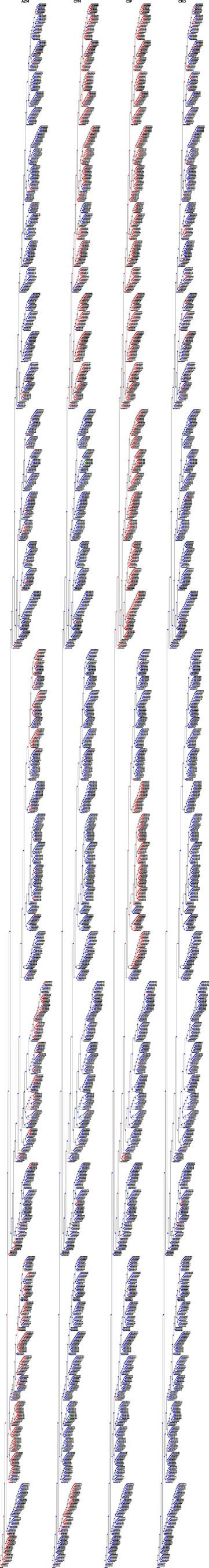
Ancestral state reconstruction of resistance categories in the *N. gonorrhoeae* RASE database. Each panel corresponds to a single antibiotic and displays the database phylogenetic tree, colored according to the reconstructed resistance categories for the antibiotic (blue, green, red, violet correspond to ‘susceptible’, ‘unknown – inferred susceptible’, ‘non-susceptible’, ‘unknown – inferred non-susceptible’, respectively).

For all antibiotics, RASE associates individual strains with a resistance category: ‘susceptible’ (S) or ‘non-susceptible’ (R). First, intervals of possible MIC values are extracted using regular expressions from the available textual antibiograms. For instance, ‘≥4’, ‘2’ and ‘NA’ would be translated to the intervals [4, + ∞), [2, 2] and [0, + ∞), respectively. Then the acquired intervals are compared to the antibiotic-specific breakpoints. If a given breakpoint is above or below the interval, susceptibility or non-susceptibility is reported, respectively. However, no category can be assigned at this step if the breakpoint lies within the extracted interval, an antibiogram is entirely missing, it is insufficiently specific or its parsing failed. Finally, missing categories are inferred using ancestral state reconstruction on the associated phylogenetic tree while maximizing parsimony (that is, minimizing the number of nodes switching its resistance category; Extended Data Figs. 3 and 4). When the solution for a node is not unique, non-susceptibility is assigned.

### Genomic neighbour typing using nanopore sequencing

All reference strains in the database are associated with similarity weights that are set to zero at the start of the run. Each time a new read is read from the stream, *k*-mer-based matching is applied to identify the strains with the maximum number of matching *k*-mers (see below). Such strains are nearest neighbours to the read in the database according to the 1/(‘number of matched *k*-mers’) pseudodistance.

The weights of the nearest neighbours are then increased according to the information content of the read, calculated as the number of matched *k*-mers divided by the number of nearest neighbours. Reads that do not match (that is, 0 matching *k*-mers in the database) are not used in subsequent analysis. The computed matches are also used for updating the *k*-mer score (KS), which is the proportion of matched *k*-mers in all reads. The KS helps to assess whether a sample is truly matching the database and whether predicting resistance for the database species makes sense.

The obtained weights serve as a proxy to the inverted genetic distance and are used as a basis for the subsequent predictions of the lineage and the antibiotic resistance and susceptibility.

### Predicting lineage

A lineage is predicted as the lineage of the best-matching reference strain; that is, the one with the largest weight. The quality of lineage prediction is further quantified using a lineage score (LS), calculated as LS = 2 *f*/(*f* + *t*) – 1, where *f* and *t* denote the weights of the best matches in the first (‘predicted’) and in the second best (‘alternative’) lineage, respectively. The values of the LS can range from 0.0 to 1.0 with the following special cases: LS = 1.0 means that all reads were perfectly matching the predicted lineage, whereas LS = 0.0 means that the predicted and alternative lineages were matched equally well.

The LS is used to measure how well a sample matches the identified lineage. If the LS is higher than a specified threshold (0.6 in default settings), the call is considered successful. If the score is lower than this, the sample cannot be securely assigned to a lineage, and this should draw the attention of the operators. Note that custom RASE databases may require a recalibration of the threshold.

### Predicting resistance and susceptibility

Resistance or susceptibility is independently predicted for individual antibiotics based on the weights of the strains that belong to the predicted lineage. These are used to calculate a susceptibility score, which is further interpreted by comparing to predefined thresholds.

The susceptibility score (SS) is calculated as SS = *s*/(*s* + *r*), where *s* and *r* denote the weights of the best-matching susceptible and best-matching non-susceptible strains within the lineage, respectively. The values of the SS can range from 0.0 to 1.0 with the following special cases: SS = 0.0 and SS = 1.0 mean that all reads match only resistant or susceptible strains in the lineage, respectively. In practice, this happens only if the lineage is entirely associated with resistance or susceptibility. SS = 0.5 means that the best matching resistant and susceptible strains are matched equally well. As follows from the score definition, if SS is greater than 0.5, then the best-matching strain is susceptible, otherwise it is non-susceptible.

The SS is used for predicting resistance or susceptibility and for evaluating the confidence of the prediction. If the SS is greater than 0.5, susceptibility to the antibiotic is reported, otherwise non-susceptibility is reported. Hence, resistance is predicted as the resistance of the best match. However, when the SS is within the [0.4, 0.6] range, it is considered a low-confidence call, and as such it should draw the attention of the operators; this usually indicates that resistance or susceptibility has recently emerged in the evolutionary history, and genomic neighbour typing may not be able to confidently distinguish between these similar, but phenotypically distinct, strains. Note that the thresholds above might require a further recalibration based on the specific database, antibiotics and application of RASE.

### *S. pneumoniae* RASE database

The *S. pneumoniae* RASE database was constructed using the EUCAST breakpoints^33^ for the following antibiotics (mg l^–1^): ceftriaxone (0.25), erythromycin (0.25), benzylpenicillin (0.06), trimethoprim-sulfamethoxazole (1.00) and tetracycline (1.00). While we used the above values in the present work, others may be readily defined and the database rapidly updated. This is especially useful in cases where breakpoints may vary depending on the site of infection (as is the case with pneumococcal meningitis and otitis media, for which lower MICs are considered to be resistant^33^).

The draft assemblies were downloaded from the SRA FTP server using the accession codes provided in table 1 in ref. ^24^. The phylogenetic tree was downloaded from DataDryad (accession no.: 10.5061/dryad.t55gq). The pneumococcal ProPhyle index was constructed with the *k*-mer size of *k* = 18.

The obtained *S. pneumoniae* RASE database, including the source code and data, is available at https://github.com/c2-d2/rase-db-spneumoniae-sparc.

### *N. gonorrhoeae* RASE database

The *N. gonorrhoeae* RASE database was constructed with the CDC GISP breakpoints^34^ for the following antibiotics (mg l^–1^): azithromycin (2.0), cefixime (0.25), ciprofloxacin (1.0) and ceftriaxone (0.125). Before applying the breakpoints, azithromycin MICs for strains collected before 2005 were doubled to correct for the known inconsistencies of the phenotyping protocol, which were due to a change in formulation of the commercial media^47^.

The draft assemblies and the phylogenetic tree were downloaded from Zenodo (accession no.: 10.5281/zenodo.2618836). The following three prevalent types of plasmids^48^ were downloaded from GenBank, localized in the GISP database using BLAST^49^ and removed from the dataset: the cryptic plasmid pJD1 (GenBank accession no.: NC_001377.1), the beta-lactamase plasmid pJD4 (GenBank accession no.: NC_002098.1) and the conjugative plasmid pEP5289 (GenBank accession no.: GU479466.1). The gonococcal ProPhyle index was constructed with the *k*-mer size of *k* = 18.

The obtained *N. gonorrhoeae* RASE database, including the source code and data, is available at https://github.com/c2-d2/rase-db-ngonorrhoeae-gisp.

### *k*-mer-based matching

Reads were matched against the RASE databases using the ProPhyle classifier^27,28^ (commit b55e026) and its ProPhex component^50,51^. The ProPhyle index stores *k*-mers of all strains in a highly compressed form, reducing the required memory footprint. In the database construction phase, the *k*-mers of the strains are first propagated along the phylogenetic tree and then greedily assembled to simplitigs^52^. The obtained simplitigs are then placed into a single text file, for which a BWT index is constructed^30^.

In the course of sequencing, each read is decomposed into overlapping *k*-mers. The *k*-mers are then searched in the BWT index by ProPhex using BWT search using a sliding window^50^. For every *k*-mer, the obtained matches are translated back on the tree. This provides a list of nodes whose descending leaves are the strains containing that *k*-mer. Finally, strains with maximum number of matched *k*-mers are identified for each read and reported in the SAM/BAM format^53^.

### Optimizing the *k*-mer length

**Extended Data Fig. 5:**
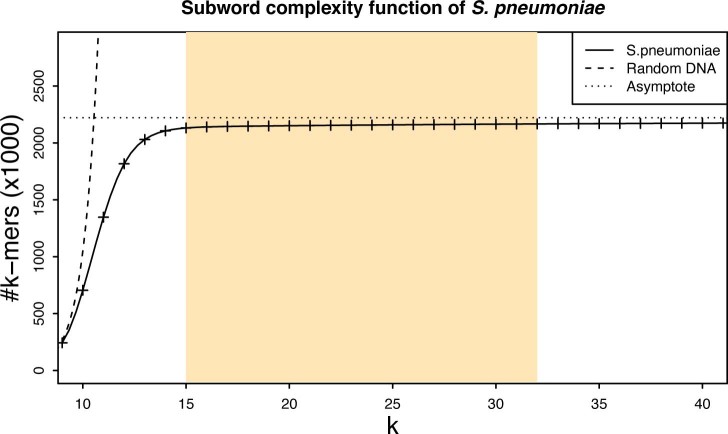
Subword complexity of pneumococcus. The plot depicts the number of canonical *k*-mers as a function of *k* for *S. pneumoniae* ATCC 700669 (GenBank accession: ‘NC_011900.1’) and for a random DNA text containing all possible *k*-mers. For *k*<10, the pneumococcus *k*-mer composition is similar to the one of random text. For *k*>14, the *k*-mer sets are almost saturated and the complexity grows very slowly. Since the genome length is finite and bacterial chromosomes are circular, the function attains its maximum at the genome size (2,221,315 in this case). The highlighted region corresponds to the range of values of *k*, which are suitable for use in RASE.

**Extended Data Fig. 6:**
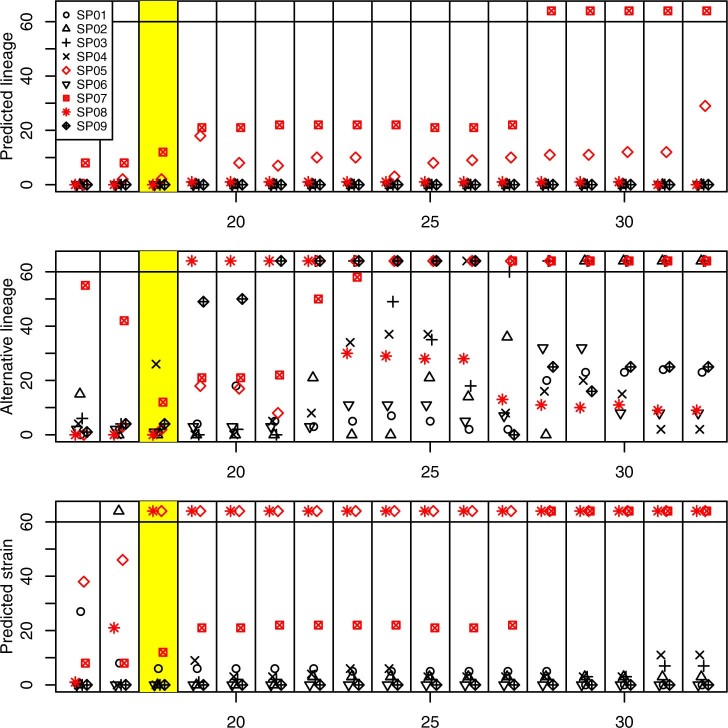
Delays in prediction based on the *k*-mer length. The plot displays delays in prediction as a function of the used *k*-mer length, for selected experiments and all possible *k*-mer lengths. Each horizontal panel displays times required for stabilization of one of the three predictions: the lineage, the alternative lineage, and the closest strain. Every column within a panel corresponds to a single *k*-mer length. When the required time exceeded 1 hour, the point is displayed at the top. Experiments where lineage could not be identified are plotted in red. The highlighted column corresponds to the *k*-mer length used for constructing the RASE databases in this paper.

The *k*-mer length is the main parameter of the classification. First, the subword complexity function^54^ of pneumococcus was calculated using JellyFish^55^ (v.2.2.10) (Extended Data Fig. 5). Then, based on the characteristics of the function and the *k*-mer range supported by ProPhyle, the possible range of *k* was determined as previously described^17,32^. For these *k*-mer lengths, RASE indexes were constructed and their performance evaluated using the RASE prediction pipeline and selected experiments. While RASE showed robustness to *k*-mer length in terms of final predictions, prediction delays differed (Extended Data Fig. 6). Based on the obtained timing data, we set the *k*-mer length to *k* *=* 18.

### Comparison to Kraken

For each RASE database, a fake NCBI taxonomy was generated from the database tree. Then, a library was built using Kraken^31^ (v.1.1.1, default parameters) from the same FASTA files as used for building the RASE database. Finally, Kraken databases were constructed for both *k* = 18 and *k* = 31.

The obtained Kraken databases were used to classify reads from individual experiments. The obtained Kraken assignments were subsequently converted using an ad hoc Python script to RASE-BAM (a subset of the BAM format^53^ used by RASE). Finally, RASE prediction was applied on the BAM files, with the use of the RASE database metadata, and the results compared with the results of the standard RASE with ProPhyle.

### Measuring time

To determine how RASE works with nanopore data generated in real time, the timestamps of individual reads were extracted using regular expressions from the read names. These were then used for sorting the base-called nanopore reads by time. When the RASE pipeline was applied, the timestamps were used for expressing the predictions as a function of time. The times of ProPhyle assignments were also compared to the original timestamps to ensure that the prediction pipeline was not slower than sequencing.

When timestamps of sequencing reads were not available (that is, the gonococcal WHO data and clinical samples), RASE estimated the progress in time from the number of processed base pairs. This was done by dividing the cumulative base-pair count by the typical nanopore flow, which we had previously estimated from SP01 as 1.43 Mbps per s. However, such an estimated progress is indicative only, as it does not follow the true order of reads in the course of sequencing. As the nanopore signal quality tends to decrease over time (see the decrease of KS in Fig. 2 after *t* = 15 mins), the randomized read order provides results of lower quality than true real-time sequencing.

### Lower time estimates on resistance gene detection

A complete genome of the multidrug-resistant SP02 isolate was assembled from the nanopore reads using CANU^56^ (v.1.5, default parameters). Before the assembly step, reads were filtered using SAMsift^57^ based on the matching quality with the pneumococcal RASE database: only reads at least 1,000-bp long with at least 10% 18-mers shared with some of the reference draft assemblies were used. The obtained assembly was further corrected using Pilon^58^ (v.1.2, default parameters) and Illumina reads from the same isolate (taxid 1QJAP in the SPARC dataset^24^) mapped to the nanopore assembly using BWA-MEM^59^ (v.0.7.17, default parameters) and sorted using SAMtools^53^.

The obtained assembly was searched for resistance-causing genes using the online CARD tool^16^ (as of 1 Aug 2018). All of the original nanopore reads were then mapped using Minimap2 (v.2.11, with ‘-x map-ont’)^60^ to the corrected assembly, and resistance genes in the reads were identified using BEDtools–intersect^61^ (v.2.27.1, with ‘-F 95’). Timestamps of the resistance-informative reads were extracted and associated with the genes. Only reads longer than 2 kbp were used in the analysis.

### Evaluation of the *N. gonorrhoeae* WHO samples

To evaluate the predictions of the WHO samples, we inferred a phylogenetic tree from a dataset comprising both the GISP isolates and the WHO isolates. First, reads were downloaded for the GISP isolates (NCBI BioProject no.: PRJEB2999 and PRJEB7904) and for the WHO isolates F–P (NCBI BioProject no.: PRJEB4024). For the WHO isolates U–Z, read data were simulated from the finished de novo assemblies (NCBI BioProject no.: PRJEB14020) using Art-Illumina^62^ (v.2.5.1). Reads were mapped to the NCCP11945 reference genome (GenBank accession no.: CP001050.1) using BWA-MEM^59^ (v.0.7.17) and deduplicated using Picard^63^ (v.2.8.0). Pilon^58^ (v.1.16, with ‘–mindepth 10–minmq 20’) was used to call variants and further filtered to include only ‘pass’ sites, and sites where the alternative allele was supported with AF > 0.9. Gubbins^64^ (v.2.3.4) with RAxML^65^ (v.8.2.10) were run on the aligned pseudogenomes to generate the final recombination-corrected phylogeny (Supplementary Data 1).

The closest relatives identified by RASE were verified using the obtained tree. For every WHO isolate, the obtained RASE prediction was compared to the closest GISP isolate on the tree.

### Library preparation

For isolates SP01–SP06, cultures were grown in Todd–Hewitt medium with 0.5% yeast extract (THY; Becton Dickinson) at 37 °C in 5% CO_2_ for 24 h. High-molecular-weight (>1 μg) genomic DNA was extracted and purified from cultures using a DNeasy Blood and Tissue kit (Qiagen). DNA concentration was measured using a Qubit fluorometer (Invitrogen). Library preparation was performed using an ONT 1D ligation sequencing kit (SQK LSK108).

For isolates SP07–SP12, library preparation was performed using an ONT Rapid Low-Input Barcoding kit (SQK-RLB001), with saponin-based host DNA depletion used for reducing the proportion of human reads. More details can be found in the original manuscript^40^.

For isolates GC01–GC05, cultures were grown on Chocolate-Agar media (that is, Difco GC base media containing 1% IsoVitaleX (Becton Dickinson) and 1% Remel Hemoglobin (Thermo Fisher Scientific)) at 37 °C in 5% CO_2_ for 20 h. For GC01–GC04, genomic DNA was extracted and purified from cultures using a PureLink Genomic DNA mini kit (Thermo Fisher Scientific). For GC05, DNA was extracted using the phenol–chloroform method^66^. Genomic DNA was extracted and purified from cultures using the PureLink Genomic DNA mini kit (Thermo Fisher Scientific). DNA concentration was measured using a Qubit fluorometer (Invitrogen). Library preparation was performed using the ONT 1D ligation sequencing kit (SQK-LSK109).

### MinION sequencing

Sequencing was performed on a MinION MK1 device using R9.4/FLO-MIN106 flow cells according to the manufacturer’s instructions. For experiments SP01–SP06, base calling was performed using ONT Metrichor (v.1.6.11 (SP01), v.1.7.3 (SP02), v.1.7.14 (SP03–SP06)) simultaneously with sequencing, and all reads passing Metrichor quality check were used in the further analysis. For experiments SP07–SP12, the ONT MinKNOW software (v.1.4-1.13.1) was used to collect raw sequencing data, and ONT Albacore (v.1.2.2-2.1.10) was used for local base-calling of the raw data after sequencing runs were completed. For experiments GC01–GC05, ONT MinKNOW software was used to collect raw sequencing data, and ONT Albacore (v.2.3.4) was used for local base-calling.

### Testing resistance phenotype

Additional retesting of SPARC isolates was done using microdilution. Organism suspensions were prepared from overnight growths on blood agar plates to the density of a 0.5 McFarland standard. This organism suspension was then diluted to provide a final inoculum of 105 to 106 colony-forming units per ml. Microdilution trays were prepared according to the NCCLS methodology with cation-adjusted Mueller–Hinton broth (Sigma-Aldrich) supplemented with 5% lysed horse blood (Hemostat Laboratories)^67,68^. Penicillin (TRC Canada) and chloramphenicol (USB) concentrations ranged from 0.016 to 16 μg ml^–1^. Erythromycin (Enzo Life Sciences), tetracycline (Sigma-Aldrich) and trimethoprim–sulfamethoxazole (MP Biomedicals) concentrations ranged from 0.0625 to 64 μg ml^–1^. Ceftriaxone (Sigma-Aldrich) concentrations ranged from 0.007 to 8 μg ml^–1^. The microdilution trays were incubated in ambient air at 35 °C for 24 h. The MICs were then visually read and breakpoints applied. A list of individual microdilution measurements and the obtained resistance categories is provided in Supplementary Table 2.

Resistance of streptococcus in the metagenomic samples (SP07–SP12) was determined by agar diffusion using the EUCAST methodology and breakpoints^33^. First, the inoculated agar plates were incubated at 37 °C overnight and then examined for growth, with the potential for reincubation up to 48 h. Then, the samples were screened to oxacillin: if the zone diameter *r* was >20 mm, the isolate was considered to be sensitive to benzylpenicillin, otherwise a full MIC measurement to benzylpenicillin was done. Finally, the isolate was screened for resistance to tetracycline (*r* ≥ 25 mm for sensitive, *r* < 22 mm for resistant) and erythromycin (*r* ≥ 22 mm for sensitive, *r* < 19 mm for resistant); when the isolate showed intermediate resistance, a full MIC measurement was done.

Results for all tested samples, isolates and metagenomes are summarized in Supplementary Table 3.

### Reporting Summary

Further information on research design is available in the Nature Research Reporting Summary linked to this article.

## Supplementary information

Supplementary Information Supplementary notes, Supplementary Table 1, Supplementary Figs. 1–3 and supplementary references.

Reporting Summary

Supplementary Table 2 Additional MIC measurements for selected strains of *S. pneumoniae*. The table displays results from strain retesting. Each record contains the date of when the retesting was done, the antibiotic, the measured MIC and the corresponding resistance category.

Supplementary Table 3 Overview of performed resistance tests for a) *S. pneumoniae* and b) *N. gonorrhoeae*. For all sequencing experiments, the table displays the best matching strains, their MICs and all measurements of database MICs (the original reported values or categories inferred using ancestral state reconstruction when not available, retested values and the resulting resistance categories).

Supplementary Table 4 Metadata for all strains included in the a) *S. pneumoniae* and b) *N. gonorrhoeae* RASE database. Each record contains the strain’s taxid, lineage, serotype (for *S. pneumoniae* only), MLST sequence type, order in the phylogenetic tree and three fields related to resistance for every antibiotics: the ‘_mic’, ‘_int’, ‘_cat’ fields contain the original published MIC information (possibly corrected after retesting), the extracted MIC interval and the resulting category after ancestral state reconstruction (S = susceptible, R = non-susceptible, s = unknown but reconstructed susceptible, r = unknown but reconstructed non-susceptible), respectively.

Supplementary Table 5 Prevalence of resistance phenotypes across lineages in the a) *S. pneumoniae* and b) *N. gonorrhoeae* RASE database. Statistics on prevalence of resistance phenotypes across lineages before and after the ancestral state reconstruction step.

Supplementary Table 6 Sensitivity and specificity of resistance and susceptibility inference in all the datasets. The table shows the number of true positive (TP), true negative (TN), false negative (FN) and false positive (FP) calls for resistance/susceptibility in individual datasets and the resulting sensitivity and specificity.

Supplementary Table 7 Comparison of ProPhyle- and Kraken-powered genomic neighbor typing. The table shows the final resistance and susceptibility inference calls for the ProPhyle (*k* = 18) and Kraken (*k* = 18 and *k* = 31) classifiers plugged into RASE.

Supplementary Data 1 Comprehensive phylogenetic tree for *N. gonorrhoeae*. A recombination-corrected tree in the Newick format comprising the GISP database strains and the WHO strains.

## Data Availability

The analyses in the paper were performed using the following RASE databases: “*N. gonorrhoeae* GISP USA v1.4” (available at https://github.com/c2-d2/rase-db-ngonorrhoeae-gisp/releases) and “*S. pneumoniae* SPARC USA v1.3” (available at https://github.com/c2-d2/rase-db-spneumoniae-sparc/releases). Nanopore reads for all experiments from this study have been deposited in Zenodo with the accession code 10.5281/zenodo.3346055; for the metagenomic experiments (SP07–SP12), only the filtered datasets were made publicly available (that is, after removing the remaining human reads in silico to comply with privacy policies). Additional supplementary materials are available at https://github.com/c2-d2/rase-supplement.

## Code availability

RASE was developed using Python, GNU Make, GNU Parallel^69^, Snakemake^70^ and the ETE 3 (ref. ^71^) and PySam^53^ libraries, and was based on ProPhyle^27,28^ (commit b55e026). Bioconda^72^ was used to ensure reproducibility of the software environments. The core RASE package and RASE documentation are available at https://github.com/c2-d2/rase. The prediction pipeline is available at https://github.com/c2-d2/rase-pipeline. Codes for constructing the *S. pneumoniae* and *N. gonorrhoeae* RASE databases are available at https://github.com/c2-d2/rase-db-spneumoniae-sparc and https://github.com/c2-d2/rase-db-ngonorrhoeae-gisp, respectively. A skeleton for creating new databases is available from https://github.com/c2-d2/rase-db-skeleton.
