# Supplementary material for: Rapid inference of antibiotic resistance and susceptibility by genomic neighbour typing
Source: Nat Microbiol. 2020 Feb 10;5(3):455–64. doi: 10.1038/s41564-019-0656-6 (PMC7044115; doi:10.1038/s41564-019-0656-6)
Supplement: Supplementary file 1 — Supplementary notes, Supplementary Table 1, Supplementary Figs. 1–3 and supplementary references. [file 41564_2019_656_MOESM1_ESM.pdf]

nature microbiology

SUPPLEMENTARY INFORMATION

https://doi.org/10.1038/s41564-019-0656-6

In the format provided by the authors and unedited.

Rapid inference of antibiotic resistance and susceptibility by genomic neighbour typing

Karel Břinda[1,2*], Alanna Callendrello[1], Kevin C. Ma[3], Derek R. MacFadden[1,4], Themoula Charalampous[5], Robyn S. Lee[1,6], Lauren Cowley[7], Crista B. Wadsworth[8], Yonatan H. Grad[3], Gregory Kucherov[9,10], Justin O'Grady[11,5], Michael Baym[2] and William P. Hanage[1]

[1]Center for Communicable Disease Dynamics, Department of Epidemiology, Harvard T. H. Chan School of Public Health, Boston, MA, USA. [2]Department of Biomedical Informatics and Laboratory of Systems Pharmacology, Harvard Medical School, Boston, MA, USA. [3]Department of Immunology and Infectious Diseases, Harvard T. H. Chan School of Public Health, Boston, MA, USA. [4]Division of Infectious Diseases, Department of Medicine, University of Toronto, Toronto, Ontario, Canada. [5]Norwich Medical School, University of East Anglia, Norwich Research Park, Norwich, UK. [6]Epidemiology Division, Dalla Lana School of Public Health, University of Toronto, Toronto, Ontario, Canada. [7]Department of Biology and Biochemistry, University of Bath, Bath, UK. [8]Thomas H. Gosnell School of Life Sciences, Rochester Institute of Technology, Rochester, NY, USA. [9]CNRS/LIGM Université Paris-Est, Marne-la-Vallée, France. [10]Skolkovo Institute of Science and Technology, Moscow, Russia. [11]Quadram Institute Bioscience, Norwich Research Park, Norwich, UK.
*e-mail: kbrinda@hsph.harvard.edu

Supplementary Information

Rapid inference of antibiotic resistance and susceptibility by genomic neighbor typing

Karel Břinda, Alanna Callendrello, Kevin C Ma, Derek R MacFadden, Themoula Charalampous, Robyn S Lee, Lauren Cowley, Crista B Wadsworth, Yonatan H Grad, Gregory Kucherov, Justin O'Grady, Michael Baym, and William P Hanage

Contents

1 Supplementary Notes **1**

2 Supplementary Tables **3**

3 Quantification of the association of bacterial clones with antibiotic resistance **4**

3.1 Introduction 4

3.2 Optimal lineage-to-resistance classifiers 4

3.3 Results 6

Bibliography **7**

1 Supplementary Notes

SI Note 1. Out of all 616 pneumococcal strains (Supplementary Table 4a), after the ancestral reconstruction step 485 were associated with susceptibility to ceftriaxone, 484 to erythromycin, 341 to benzylpenicillin, 480 to trimethoprim-sulfamethoxazole, and 551 to tetracycline (Supplementary Table 5a). In case of gonococcus, ancestral reconstruction was needed only for cefixime (62 records affected). Out of all 1102 gonococcal strains (Supplementary Table 4b), 808 were associated with susceptibility to azithromycin, 833 to cefixime, 508 to ciprofloxacin, and 1033 to ceftriaxone (Supplementary Table 5b). In our subsequent experiments, if original MIC data were not available for the best match in the RASE database, the relevant strain was tested to confirm resistance phenotype (Methods).

SI Note 2. We evaluated how long it took for resistance genes to be reliably detected in nanopore reads. For SP02 we observed that at least 25 minutes were needed to detect resistance (i.e., to observe all resistance genes at least once), assuming that the genes in question can be unambiguously identified in nanopore data despite the high per-base error rate, and that the presence of the loci is directly linked to the resistance phenotype (Extended Data Fig. 2). If this is not the case (for example if resistance is conferred by a single SNP, requiring coverage with multiple reads), further delays would be expected. Thus, genomic neighbor typing can offer a time advantage compared to methods based on identifying the presence of resistance genes even in a sample of DNA from a purified isolate as opposed to a metagenome, potentially allowing for more rapid changes to antimicrobial therapy.

SI Note 3. We originally attempted to evaluate a multidrug-resistant isolate (GCGS0938 in the GISP collection); however, RASE placed it onto a distant part of the phylogeny and identified it as GCGS0324 or GCGS1095. A subsequent analysis revealed that the sample was mislabeled and that it was indeed GCGS1095, i.e., the same strain as in GC02, although from a different stock.

SI Note 4. We evaluated how RASE performs in extremely unfavorable sequencing conditions; we sequenced an isolate (GC05) from the GISP collection with the use of an expired flow cell (purchased in October 2017, expired in December 2017, and the sequencing done in April 2018). In consequence, we obtained only 3.5 Mbps of low-quality reads (only 7% of matching k-mers compared to 20% obtained in the other isolates)

(GC05 in Table 2a). An experiment with such a low yield would normally be discarded; despite that RASE provided correct and stabilized predictions (once the first long read was obtained from the sequencer at t=21mins).

SI Note 5. We evaluated how genomic neighbor typing would perform if RASE used Kraken (Wood and Salzberg 2014) instead of ProPhyle (Břinda et al. 2017) for the read-to-strain comparison (the matching step in Fig. 1). Both tools use k-mer-based matching to assign sequencing reads to a phylogenetic tree, but with several key differences. Whereas Kraken stores for each k-mer the lowest common ancestor (LCA) only, assigns reads to the LCA of the best hits and ignores low-complexity k-mers, ProPhyle indexes all k-mers using an exact index and can thus resolve ambiguities both on the level of individual k-mers and read assignments.

To compare both tools, we implemented a RASE wrapper for Kraken (Methods) and applied that to the same read and database data. We then compared the final inference results obtained with Kraken (with k=18 and k=31) with the results obtained from the standard RASE pipeline (Supplementary Table 7).

For *S. pneumoniae* and *N. gonorrhoeae*, the number of inference errors increased more than 1.5x and 1.7x, respectively (in case of both k-mer sizes). In the case of *N. gonorrhoeae*, RASE-Kraken showed large systematic biases in neighbor typing, assigning 16 (k=18) and 18 (k=31) out of the gonococcal 33 samples to a single strain (GCGS1028), whereas RASE-ProPhyle identified this strain only once. While in the WHO dataset the numbers of RASE-ProPhyle and RASE-Kraken errors were comparable (10 vs. 12 and 11), in the RaDAR-Go dataset it increased from 1 to 8 and 10. Overall, the obtained results suggest that Kraken is less suited for the use in genomic neighbor typing than ProPhyle.

SI Note 6. We analyzed the results of the WHO gonococcal samples (Supplementary Table 1). First, we evaluated the RASE ability to predict MLST sequence types. In all cases, either RASE predicted the correct sequence type (n=9), or the true sequence type was not present in the reference database (n=5). The latter was the case only in the samples F through P, which belonged to the initial 2008 WHO reference panel and were collected primarily in the late 1990s, with the majority of specimens isolated from the Eastern Hemisphere (Unemo et al. 2009). The GISP database, comprising strains collected in the US from 2000–2013, may not be representative then of the circulating lineages in those regions during that time span, which could result in both sequence type and antibiogram prediction errors. However, we observed perfect prediction of sequence types in the additional 2016 WHO reference strains comprising U through Z that were collected in 2007 and onwards (Unemo et al. 2016).

We next sought to evaluate the resistance predictions. In 7 cases (F, K, N, O, P, U, W), the antibiograms were identified fully correctly; in 4 (G, V, X, Z) and 3 cases (L, M, Y) one and two mistakes were made, respectively. To explain these discrepancies, we inferred a recombination-corrected phylogenetic tree comprising the GISP database isolates as well as the WHO samples (Supplementary Data 1). With the exception of G and Y, the WHO isolates and their respective RASE-predicted best matches were the closest GISP isolates, indicative of accurate matching by RASE. While branch lengths of L, M and V on the tree reveal that the corresponding parts of the phylogeny are not well sampled in the database, the X, Y, and Z samples emerged from lineages that are well-represented but have acquired an atypically high level of cephalosporin resistance. Whereas X and Z acquired a novel resistance-conferring mosaic penA allele (Ohnishi et al. 2011), Y acquired a novel active site mutation in the context of a pre-existing mosaic penA allele (Unemo et al. 2011). While both of these adaptations resulted in high-level resistance, these mutations also appear to incur fitness costs in vitro and in the gonococcal mouse model (Vincent et al. 2018). In line with this, these strains have only been sporadically observed in genomic surveillance of clinical isolates. These results highlight how ancestral or emerging resistant lineages may not be well-captured by sequence-based methods including RASE and emphasize the value of continuous updating of the RASE database for public health.

SI Note 7. Further analysis of the reads from SP12 using Krocus (Page and Keane 2018) suggested that the pneumococcal DNA present was from the ST180 clonal complex, and matched specifically either to the sequence type ST180 or ST3798. This is consistent with identification as serotype 3, because this clonal complex contains the great majority of strains with this capsule type, which historically has not been associated with resistance (Nicholas J. Croucher et al. 2013). However, improved sampling and study of this lineage has recently found highly divergent subclades that are associated with resistance. These lineages were

previously rare, and thus were less likely to be included in our database, but now are increasing in frequency (Azarian et al. 2018). In this case, ST3798 is found to be in clade 1B, which is notable for exhibiting sporadic tetracycline resistance. Again, the failure to match to this is a result of the original database not containing a suitable example for comparison.

SI Note 8. The suitability of genomic neighbor typing for a specific species-drug combination is a result of complex tradeoffs, which ultimately must be tested empirically. Generally speaking, the method relies on the resistance phenotype being correlated with the variation across the genome. In practice, this means that any situation reducing this correlation would limit the application. This includes too little within-species genomic variation, as this would lower the precision of nearest neighbor identification. Another example is resistance emerging rapidly on independent and diverse genomic backgrounds breaking down the correlation. Overall, the criteria for suitability of genomic neighbor typing are a fruitful subject for future research/

2 Supplementary Tables

Supplementary Table 1. Predicted phenotypes of *N. gonorrhoeae* for the WHO strains. Predicted phenotypes of *N. gonorrhoeae* for the WHO strains. The table displays actual and predicted resistance phenotypes (S = susceptible, R = non-susceptible) for individual experiments, as well as information on match of the predicted MLST sequence type.

Supplementary Table 1. Predicted phenotypes of *N. gonorrhoeae* for the WHO strains

Sample	Region	Lineage confidently detected	Matched k-mers	Antibiogram AZM		Antibiogram CFM		Antibiogram CIP		Antibiogram CRO		MLST match
				Actual	Best match	Actual	Best match	Actual	Best match	Actual	Best match	
WHO F (2008)	Canada	no	17%	S	S!	S	S	S	S	S	S	OoD
WHO G (2008)	Thailand	no	14%	S	S	S	S	S	R	S	S	OoD
WHO K (2008)	Japan	yes	20%	S	S	R	R	R	R	S	S	yes
WHO L (2008)	Asia	yes	20%	S	S	S	R	R	S	R	R	OoD
WHO M (2008)	Philippines	yes	21%	S	R	S	S	R	S	S	S	yes
WHO N (2008)	Australia	no	19%	S	S	S	S	R	R	S	S	OoD
WHO O (2008)	Canada	yes	20%	S	S	S	S	S	S	S	S	yes
WHO P (2008)	USA	yes	19%	R	R	S	S	S	S	S	S	OoD
WHO U (2016)	Sweden	yes	20%	R	R	S	S	S	S	S	S	yes
WHO V (2016)	Sweden	yes	19%	R	S	S	S	R	R	S	S	yes
WHO W (2016)	Hong Kong	yes	20%	S	S	R	R	R	R	S	S	yes
WHO X (2016)	Japan	yes	21%	S	S	R	R	R	R	R	S	yes
WHO Y (2016)	France	no	18%	S	S	R	S	R	R	R	S	yes
WHO Z (2016)	Australia	yes	19%	S	S	R	R	R	R	R	S	yes

Legend

Colour		Symbol	Meaning
Correct prediction (green)		S	Susceptible
Incorrect prediction (orange)		R	Non-susceptible
Cannot be evaluated (purple)		!	Low-confidence call
		OoD	Out-of-database

Supplementary Table 2. Additional MIC measurements for selected strains of *S. pneumoniae*. The table displays results from strain retesting. Each record contains date when the retesting was done, the antibiotic, the measured MIC, and the corresponding resistance category.

Supplementary Table 3. Overview of performed resistance tests for a) *S. pneumoniae* and b) *N. gonorrhoeae*. For all sequencing experiments, the table displays the best matching strains, their MICs, and all measurements of database MICs (the original reported values or categories inferred using ancestral state reconstruction when not available, retested values, and the resulting resistance categories).

Supplementary Table 4. Metadata for all strains included in the a) *S. pneumoniae* and b) *N. gonorrhoeae* RASE database. Each record contains the strain's taxid, lineage, serotype (for *S. pneumoniae* only), MLST sequence type, order in the phylogenetic tree, and three fields related to resistance for every antibiotics: the '_mic', '_int', '_cat' fields contain the original published MIC information (possibly corrected after retesting), the extracted MIC interval, and the resulting category after ancestral state reconstruction (S = susceptible, R = non-susceptible, s = unknown but reconstructed susceptible, r = unknown but reconstructed non-susceptible), respectively.

Supplementary Table 5. Prevalence of resistance phenotypes across lineages in the a) *S. pneumoniae* and b) *N. gonorrhoeae* RASE database. Statistics on prevalence of resistance phenotypes across lineages before and after the ancestral state reconstruction step.

Supplementary Table 6. Sensitivity and specificity of resistance and susceptibility inference in all the datasets. The table shows the number of true positive (TP), true negative (TN), false negative (FN), and false positive (FP) calls for resistance/susceptibility in individual datasets and the resulting sensitivity and specificity.

Supplementary Table 7. Comparison of ProPhyle- and Kraken-powered genomic neighbor typing. The table shows the final resistance and susceptibility inference calls for the ProPhyle (k=18) and Kraken (k=18 and k=31) classifiers plugged into RASE.

3 Quantification of the association of bacterial clones with antibiotic resistance

3.1 Introduction

In this document, we show how to quantify the association of bacterial clones with antibiotic resistance. For a given bacterial species and all antibiotics of interest, we construct optimal predictors of resistance from lineages and calculate the associated Receiver Operating Characteristics (ROC) curve and its Areas under the Curve (AUC). Comparing the resulting curves and areas among antibiotics helps to understand the different levels of associations.

We use this framework to show that for the pathogens *Streptococcus pneumoniae* and *Neisseria gonorrhoeae* antibiotic resistance is highly associated with the population structure. This provides evidence of the suitability of genomic neighbor typing as a diagnostic method for these pathogens.

3.2 Optimal lineage-to-resistance classifiers

3.2.1 Model

Let us consider a bacterial species and assume that it has g lineages. For the purpose of this document, lineages are arbitrary classes of equivalence; they can be defined based on sequence typing (e.g., MLST (Maiden 2006)) or clustering (e.g., using BAPS (Cheng et al. 2013) or PopPUNK (Lees et al. 2019)). Assume that lineages are equally probable; i.e., a randomly drawn isolate x comes from every lineage i with the probability $\frac{1}{g}$. Assume that for every isolate x, we can always correctly determine its lineage $\ell(x)$. In a clinical setting, this could mean that we can always determine isolate's MLST sequence type.

Let us consider an antibiotic and assume that every isolate is either resistant or susceptible to this antibiotic. Assume that resistance within a lineage i is iid with the Bernoulli distribution and let r_i denote the probability of resistance. The constants r_i can be determined based on epidemiological data or as proportions of resistance isolates in individual lineages from population-level studies.

3.2.2 Lineage-to-resistance classifiers

For a given species, an antibiotic and fixed probabilities of resistance within lineages $r_1, \ldots, r_g$, we construct memoryless probabilistic resistance classifiers C of the form

$$C(\ell(x)) \to \{\text{'S'}, \text{'R'}\}.$$

In other words, for every isolate we identify its lineage and the classifier predicts resistance based on the knowledge of the lineage.

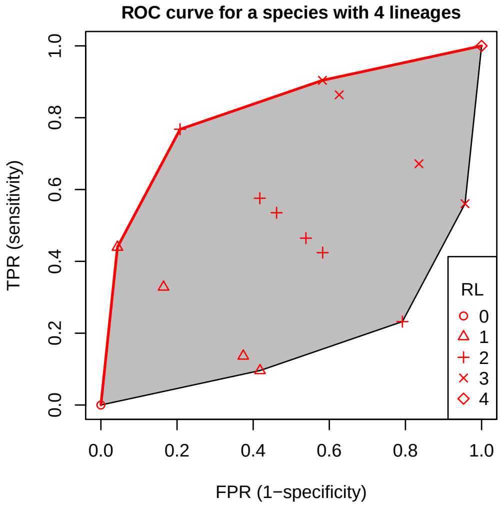

SI Fig. 1: An illustration of the ROC diagram for a species with 4 lineages with resistance probabilities $(r_i)_{i=1}^4 = (0.1, 0.4, 0.8, 0.9)$. The grey area corresponds to all the possible lineage-to-resistance classifiers $C_{(R_1,R_2,R_3,R_4)}$ while the red points are the "vertex" classifiers with integer parameters; i.e., $R_i \in \{0,1\}$ for every $i \in \{1,\ldots,g\}$. RL denotes the number of lineages for which resistance is reported by an integer classifier, i.e., $\sum_i R_i$. The optimal classifiers that maximize the AUC lie on the piece-wise linear red curve, which we term the optimal ROC curve.

All such classifiers can be parametrized as $C_{(R_1,\ldots,R_g)}$, where $R_i \in [0,1]$ is the probability of reporting resistance given the sample has been identified as belong to the lineage i. For instance, the classifier $C_{(0,\ldots,0)}$ always assigns 'S', $C_{(\frac{1}{2},\ldots,\frac{1}{2})}$ assigns 'R' and 'S' like a fair coin, $C_{(1,\ldots,1)}$ always assigns 'R', and $C_{(1,0,\ldots,0)}$ always assigns 'R' for the first lineage and 'S' for the other ones.

With the knowledge of the probabilities of resistance $(r_1,\ldots,r_g)$ within individual lineages $1,\ldots,g$, we can express false positive rate (FPR) and true positive rate (TPR) as a function of the classifier parameters $(R_1,\ldots,R_g)$.

Let us use the standard notation:

$$\mathrm{FPR} = \frac{\mathrm{FP}}{\mathrm{FP}+\mathrm{TN}} \qquad \text{and} \qquad \mathrm{TPR} = \frac{\mathrm{TP}}{\mathrm{TP}+\mathrm{FN}}$$

where

$$\begin{aligned}
\mathrm{TP} &= \#\text{true positives; i.e., resistant isolates predicted as resistant} \\
\mathrm{FP} &= \#\text{false positives; i.e., susceptible isolates predicted as resistant} \\
\mathrm{FN} &= \#\text{false negatives; i.e., resistant isolates predicted as susceptible} \\
\mathrm{TN} &= \#\text{true negatives; i.e., susceptible isolates predicted as susceptible}
\end{aligned}$$

Given our assumptions, we can estimate the performance of a classifier $C_{R_1,\ldots,R_g}$ as

$$\begin{array}{ll}
\mathrm{TP} \approx \frac{N}{g}\sum_{i=1}^g r_i R_i & \qquad \mathrm{FP} \approx \frac{N}{g}\sum_{i=1}^g (1-r_i)R_i \\
\mathrm{FN} \approx \frac{N}{g}\sum_{i=1}^g r_i(1-R_i) & \qquad \mathrm{TN} \approx \frac{N}{g}\sum_{i=1}^g (1-r_i)(1-R_i)
\end{array}$$

where N is the number of samples tested. We then obtain the following estimates:

$$\mathrm{FPR} \approx \frac{\sum_{i=1}^g (1-r_i)R_i}{\sum_{i=1}^g (1-r_i)} \qquad \text{and} \qquad \mathrm{TPR} \approx \frac{\sum_{i=1}^g r_i R_i}{\sum_{i=1}^g r_i}$$

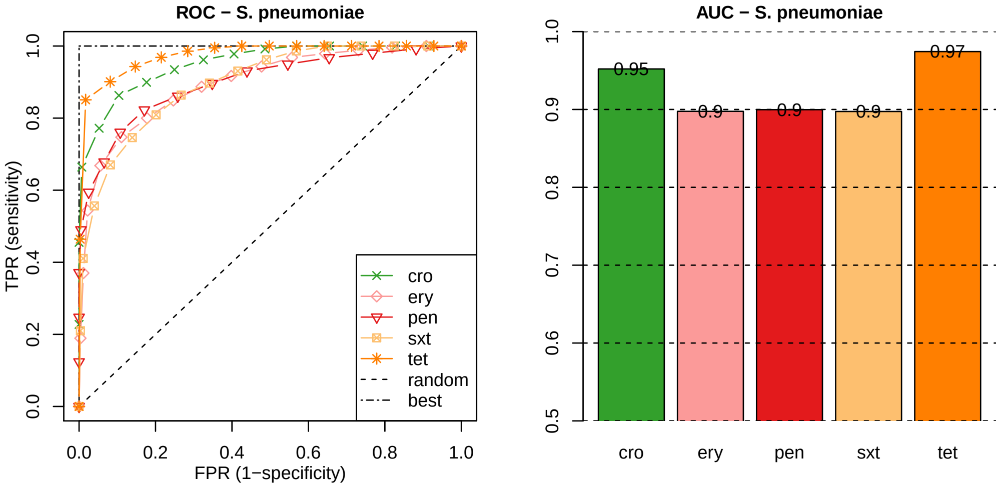

SI Fig. 2: *S. pneumoniae* ROC curves and the corresponding AUCs for ceftriaxone (cro), erythromycin (ery), penicillin (pen), trimethoprim (sxt), and tetracycline (tet).

Using these formulae, we can map every classifier $C_{(R_1,\ldots,R_g)}$ to the ROC space by

$$f(R_1,\ldots,R_g) \to \left(\frac{\sum_{i=1}^{g}(1-r_i)R_i}{\sum_{i=1}^{g}(1-r_i)}, \frac{\sum_{i=1}^{g} r_i R_i}{\sum_{i=1}^{g} r_i} \right)$$

It is easy to see that the map f is linear. Since the set of all possible classifiers is the cube $[0,1]^g$ in the parameter space, it is convex and its image in the ROC space must be convex too; an example is provided in SI Fig.~1. Moreover, the image of the cube is equal to the convex hull of the images of individual cube vertices (red points in SI Fig.~1).

3.2.3 Optimal ROC curves

Our aim now is to find the optimal ROC curve maximizing the AUC (the red curve in SI Fig.~1). Even though the curve corresponds to infinitely many classifiers, it is a piece-wise linear function which is fully defined by the $g+1$ "vertex'' classifiers $C^{(0)},\ldots,C^{(g)}$ lying on the line intersections. Enumerating this classifier sequence corresponds to putting lineages to the order in which they are switched from susceptible to resistant (i.e., $R_i = 0 \to R_i = 1$) along the red line. The first classifier $C^{(0)}$, $(0,0)$ in the ROC diagram, corresponds to all lineages being marked as susceptible, while the last classifier, $C^{(g)}$, $(1,1)$ in the diagram to all lineages being predicted marked as resistant.

The optimal ROC curve can be computed in multiple different ways. For instance, we can enumerate all cube vertices in the parameter space, map them to the ROC space, compute the convex hull, and extract its upper part. More efficiently, we can construct the classifier sequence directly in the ROC diagram by the following iterative process. We start at $(0,0)$; i.e., with all lineages marked as susceptible, and at every step we switch one susceptible lineage to resistant so that the corresponding step in the ROC graph has maximal possible slope, and we continue until all lineages are marked as resistant. If lineages are equally probable, it is easy to see that this order corresponds to sorting lineages by r_i.

3.3 Results

We applied the method to 616 pneumococcal genomes from a carriage study in Massachusetts children (Nicholas J Croucher et al. 2013; Croucher et al. 2015) and 1102 clinical gonococcal isolates collected from 2000 to 2013 by the Centers for Disease Control and Prevention's Gonococcal Isolate Surveillance Project

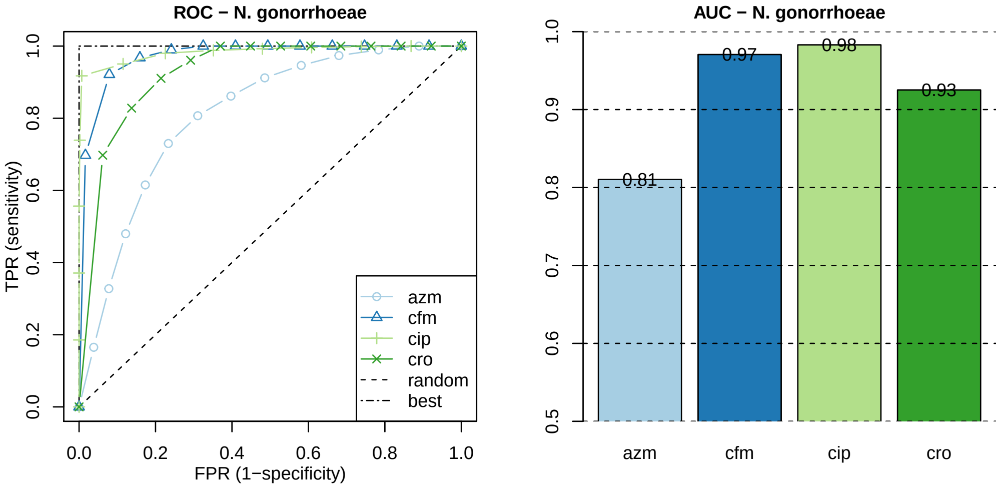

SI Fig. 3: *N. gonorrhoeae* ROC curves and the corresponding AUCs for azithromycin (azm), cefixime (cfm), ciprofloxacine (cip), and ceftriaxone (cro).

(GISP) (Grad et al. 2016). For all isolates, we inferred the resistance categories as described in the main manuscript, including the ancestral state responstruction step. As lineages we used the sequenced clusters computed using Bayesian Analysis of Population Structure (BAPS) (Cheng et al. 2013).

Lineages of *S. pneumoniae* are predictive for benzylpenicillin, ceftriaxone, trimethoprim-sulfamethoxazole, erythromycin, and tetracycline resistance with AUC ranging from 0.90 to 0.97 (SI Fig. 2). In *N. gonorrhoeae* ciprofloxacin, ceftriaxone, and cefixime attained comparably large AUCs (from 0.93 to 0.98) whereas azithromycin demonstrated lower association (AUC 0.80) (SI Fig. 3).

Bibliography

Azarian, Taj, Patrick K. Mitchell, Maria Georgieva, Claudette M. Thompson, Amel Ghouila, Andrew J. Pollard, Anne von Gottberg, et al. 2018. "Global Emergence and Population Dynamics of Divergent Serotype 3 CC180 Pneumococci." Edited by Timothy J. Mitchell. *PLOS Pathogens* 14 (11): e1007438. https://doi.org/10.1371/journal.ppat.1007438.

Břinda, Karel, Kamil Salikhov, Simone Pignotti, and Gregory Kucherov. 2017. "Karel-Brinda/Prophyle: Prophyle 0.3.0.3." Zenodo. https://doi.org/10.5281/ZENODO.1045429.

Cheng, L., T. R. Connor, J. Siren, D. M. Aanensen, and J. Corander. 2013. "Hierarchical and Spatially Explicit Clustering of DNA Sequences with BAPS Software." *Molecular Biology and Evolution* 30 (5): 1224–8. https://doi.org/10.1093/molbev/mst028.

Croucher, Nicholas J, Jonathan A Finkelstein, Stephen I Pelton, Patrick K Mitchell, Grace M Lee, Julian Parkhill, Stephen D Bentley, William P Hanage, and Marc Lipsitch. 2013. "Population Genomics of Post-Vaccine Changes in Pneumococcal Epidemiology." *Nature Genetics* 45 (6): 656–63. https://doi.org/10.1038/ng.2625.

Croucher, Nicholas J., Jonathan A. Finkelstein, Stephen I. Pelton, Julian Parkhill, Stephen D. Bentley, Marc Lipsitch, and William P. Hanage. 2015. "Population Genomic Datasets Describing the Post-Vaccine Evolutionary Epidemiology of Streptococcus Pneumoniae." *Scientific Data* 2 (1). https://doi.org/10.1038/sdata.2015.58.

Croucher, Nicholas J., Andrea M. Mitchell, Katherine A. Gould, Donald Inverarity, Lars Barquist, Theresa Feltwell, Maria C. Fookes, et al. 2013. "Dominant Role of Nucleotide Substitution in the Diversification

of Serotype 3 Pneumococci over Decades and During a Single Infection." Edited by Olivier Tenaillon. *PLoS Genetics* 9 (10): e1003868. https://doi.org/10.1371/journal.pgen.1003868.

Grad, Yonatan H., Simon R. Harris, Robert D. Kirkcaldy, Anna G. Green, Debora S. Marks, Stephen D. Bentley, David Trees, and Marc Lipsitch. 2016. "Genomic Epidemiology of Gonococcal Resistance to Extended-Spectrum Cephalosporins, Macrolides, and Fluoroquinolones in the United States, 20002013." *Journal of Infectious Diseases* 214 (10): 1579–87. https://doi.org/10.1093/infdis/jiw420.

Lees, John A., Simon R. Harris, Gerry Tonkin-Hill, Rebecca A. Gladstone, Stephanie W. Lo, Jeffrey N. Weiser, Jukka Corander, Stephen D. Bentley, and Nicholas J. Croucher. 2019. "Fast and Flexible Bacterial Genomic Epidemiology with PopPUNK." *Genome Research* 29 (2): 304–16. https://doi.org/10.1101/gr.241455.118.

Maiden, Martin C. J. 2006. "Multilocus Sequence Typing of Bacteria." *Annual Review of Microbiology* 60 (1): 561–88. https://doi.org/10.1146/annurev.micro.59.030804.121325.

Ohnishi, Makoto, Daniel Golparian, Ken Shimuta, Takeshi Saika, Shinji Hoshina, Kazuhiro Iwasaku, Shu-ichi Nakayama, Jo Kitawaki, and Magnus Unemo. 2011. "Is Neisseria Gonorrhoeae Initiating a Future Era of Untreatable Gonorrhea?: Detailed Characterization of the First Strain with High-Level Resistance to Ceftriaxone." *Antimicrobial Agents and Chemotherapy* 55 (7): 3538–45. https://doi.org/10.1128/aac.00325-11.

Page, Andrew J., and Jacqueline A. Keane. 2018. "Rapid Multi-Locus Sequence Typing Direct from Uncorrected Long Reads Using Krocus." *PeerJ* 6 (July): e5233. https://doi.org/10.7717/peerj.5233.

Unemo, Magnus, Daniel Golparian, Robert Nicholas, Makoto Ohnishi, Anne Gallay, and Patrice Sednaoui. 2011. "High-Level Cefixime- and Ceftriaxone-Resistant Neisseria Gonorrhoeae in France: NovelpenAMosaic Allele in a Successful International Clone Causes Treatment Failure." *Antimicrobial Agents and Chemotherapy* 56 (3): 1273–80. https://doi.org/10.1128/aac.05760-11.

Unemo, Magnus, Daniel Golparian, Leonor Sánchez-Bu ó, Yonatan Grad, Susanne Jacobsson, Makoto Ohnishi, Monica M. Lahra, et al. 2016. "The Novel 2016 WHONeisseria Gonorrhoeaereference Strains for Global Quality Assurance of Laboratory Investigations: Phenotypic, Genetic and Reference Genome Characterization." *Journal of Antimicrobial Chemotherapy* 71 (11): 3096–3108. https://doi.org/10.1093/jac/dkw288.

Unemo, M., O. Fasth, H. Fredlund, A. Limnios, and J. Tapsall. 2009. "Phenotypic and Genetic Characterization of the 2008 WHO Neisseria Gonorrhoeae Reference Strain Panel Intended for Global Quality Assurance and Quality Control of Gonococcal Antimicrobial Resistance Surveillance for Public Health Purposes." *Journal of Antimicrobial Chemotherapy* 63 (6): 1142–51. https://doi.org/10.1093/jac/dkp098.

Vincent, Leah R., Samuel R. Kerr, Yang Tan, Joshua Tomberg, Erica L. Raterman, Julie C. Dunning Hotopp, Magnus Unemo, Robert A. Nicholas, and Ann E. Jerse. 2018. "In Vivo-Selected Compensatory Mutations Restore the Fitness Cost of MosaicpenAAlleles That Confer Ceftriaxone Resistance inNeisseria Gonorrhoeae." Edited by Michael S. Gilmore. *mBio* 9 (2). https://doi.org/10.1128/mbio.01905-17.

Wood, Derrick E, and Steven L Salzberg. 2014. "Kraken: Ultrafast Metagenomic Sequence Classification Using Exact Alignments." *Genome Biology* 15 (3): R46. https://doi.org/10.1186/gb-2014-15-3-r46.
