# Supplementary material for: Rapid inference of antibiotic resistance and susceptibility by genomic neighbour typing
Source: Nat Microbiol. 2020 Feb 10;5(3):455–64. doi: 10.1038/s41564-019-0656-6 (PMC7044115; doi:10.1038/s41564-019-0656-6)
Supplement: Supplementary file 2 — Reporting Summary [file 41564_2019_656_MOESM2_ESM.pdf]

nature research

Corresponding author(s): Karel Břinda

Last updated by author(s): Nov 12, 2019

Reporting Summary

Nature Research wishes to improve the reproducibility of the work that we publish. This form provides structure for consistency and transparency in reporting. For further information on Nature Research policies, see Authors & Referees and the Editorial Policy Checklist.

Statistics

For all statistical analyses, confirm that the following items are present in the figure legend, table legend, main text, or Methods section.

n/a	Confirmed	
☐	☒	The exact sample size (n) for each experimental group/condition, given as a discrete number and unit of measurement
☐	☒	A statement on whether measurements were taken from distinct samples or whether the same sample was measured repeatedly
☒	☐	The statistical test(s) used AND whether they are one- or two-sided<br>*Only common tests should be described solely by name; describe more complex techniques in the Methods section.*
☒	☐	A description of all covariates tested
☒	☐	A description of any assumptions or corrections, such as tests of normality and adjustment for multiple comparisons
☒	☐	A full description of the statistical parameters including central tendency (e.g. means) or other basic estimates (e.g. regression coefficient) AND variation (e.g. standard deviation) or associated estimates of uncertainty (e.g. confidence intervals)
☒	☐	For null hypothesis testing, the test statistic (e.g. F, t, r) with confidence intervals, effect sizes, degrees of freedom and P value noted<br>*Give P values as exact values whenever suitable.*
☒	☐	For Bayesian analysis, information on the choice of priors and Markov chain Monte Carlo settings
☒	☐	For hierarchical and complex designs, identification of the appropriate level for tests and full reporting of outcomes
☒	☐	Estimates of effect sizes (e.g. Cohen's d, Pearson's r), indicating how they were calculated

Our web collection on statistics for biologists contains articles on many of the points above.

Software and code

Policy information about availability of computer code

Data collection	wget, curl
Data analysis	ONT data processing: ONT Metrichor (versions 1.6.11 (SP01), 1.7.3 (SP02), 1.7.14 (SP03–SP06)); ONT MinKNOW (versions 1.4-1.13.1) and ONT Albacore (versions 1.2.2-2.1.10) for SP07-SP12. ONT MinKNOW and Albacore (version 2.3.4) for GC01– GC05.<br><br>Other tools: ProPhyle (commit b55e026), GNU Make (version 4.2.1), GNU Parallel (version 20190322), Snakemake (version 5.4.5), ETE3 (version 3.1.1), PySAM (version 0.15.2), SAMtools (version 1.9), JellyFish (version 2.2.10), Kraken (version 1.1.1), CANU (version 1.5), Pilon (versions 1.2 and 1.16), BWA-MEM (version 0.7.17), CARD tool (as of 2018/08/01), BEDtools–intersect (version 2.27.1), Art-Illumina (version 2.5.1), Picard (version 2.8.0), Pilon (version 1.16), Gubbins (vresion 2.3.4), RAxML (version 8.2.10), and R (version 3.5.1).

For manuscripts utilizing custom algorithms or software that are central to the research but not yet described in published literature, software must be made available to editors/reviewers. We strongly encourage code deposition in a community repository (e.g. GitHub). See the Nature Research guidelines for submitting code & software for further information.

Data

Policy information about availability of data

All manuscripts must include a data availability statement. This statement should provide the following information, where applicable:
- Accession codes, unique identifiers, or web links for publicly available datasets
- A list of figures that have associated raw data
- A description of any restrictions on data availability

Sequencing data for all experiments from this study can be downloaded from http://doi.org/10.5281/zenodo.3346055. Other supplementary materials are available from https://github.com/c2-d2/rase-supplement/. The pneumococcal and gonococcal RASE databases are available from https://github.com/c2-d2/rase-db-spneumoniae-sparc and https://github.com/c2-d2/rase-db-ngonorrhoeae-gisp.

Field-specific reporting

Please select the one below that is the best fit for your research. If you are not sure, read the appropriate sections before making your selection.

☒ Life sciences ☐ Behavioural & social sciences ☐ Ecological, evolutionary & environmental sciences

For a reference copy of the document with all sections, see nature.com/documents/nr-reporting-summary-flat.pdf

Life sciences study design

All studies must disclose on these points even when the disclosure is negative.

Sample size	Sample size does not affect the statistical validity of a conclusion. The RASE databases were constructed from 616 isolates (SP) and 1102 isolates (GC). We evaluated the method with 12 (SP) and 33 (GC) sequencing experiments, where individual experiments were selected in such a way that we could test the method in several different scenarios (database isolates, non-database isolates with a representative database, non-database isolates with a non-representative database, and metagenomes).
Data exclusions	No reference strains were excluded from the databases. No sequencing experiments were excluded from the analysis.
Replication	No attempts at replication of individual sequencing experiments have been done.
Randomization	There were no experimental groups
Blinding	There were no experimental groups

Reporting for specific materials, systems and methods

We require information from authors about some types of materials, experimental systems and methods used in many studies. Here, indicate whether each material, system or method listed is relevant to your study. If you are not sure if a list item applies to your research, read the appropriate section before selecting a response.

Materials & experimental systems

n/a	Involved in the study	
☒	☐	Antibodies
☒	☐	Eukaryotic cell lines
☒	☐	Palaeontology
☒	☐	Animals and other organisms
☒	☐	Human research participants
☒	☐	Clinical data

Methods

n/a	Involved in the study	
☒	☐	ChIP-seq
☒	☐	Flow cytometry
☒	☐	MRI-based neuroimaging
